# Prevalence and Factors Associated With Caesarean Delivery in Nepal: Evidence From a Nationally Representative Sample

**DOI:** 10.7759/cureus.20326

**Published:** 2021-12-10

**Authors:** Pranta Das, Nandeeta Samad, Ashmita Sapkota, Hasan Al-Banna, Nor Azlina A Rahman, Rahnuma Ahmad, Mainul Haque, Brian Godman

**Affiliations:** 1 Statistics, University of Dhaka, Dhaka, BGD; 2 Public Health, North South University, Dhaka, BGD; 3 Public Health, School of Health and Allied Science, Pokhara University, Pokhara, NPL; 4 Social Welfare, Institute of Social Welfare and Research, University of Dhaka, Dhaka, BGD; 5 Community Medicine, International Islamic University, Kuantan, MYS; 6 Physiology, Medical College for Women and Hospital, Dhaka, BGD; 7 Pharmacology and Therapeutics, National Defence University of Malaysia, Kuala Lumpur, MYS; 8 Centre of Medical and Bio-allied Health Sciences Research, Ajman University, Ajman, ARE; 9 Pharmacoepidemiology, Strathclyde Institute of Pharmacy and Biomedical Sciences, Glasgow, GBR

**Keywords:** health survey, demographic, associated factors, nepal, prevalence, caesarian section

## Abstract

Background

Caesarian sections (CS) are life-saving management for a pregnant mother and fetus subject to obstetric complications. The World Health Organization (WHO) expected CS rates not to exceed 10 to 15 per 100 live births in any country. This study aimed to assess the prevalence of CS and its associated factors from the 2016 Nepal Demographic and Health Survey (NDHS), building on previous studies mentioned in detail in the latter part of the paper.

Methods

This study analyzed the secondary data from the 2016 Nepal Demographic and Health Survey (NDHS), conducted from June 19, 2016, to January 31, 2017. The survey is undertaken every five years; consequently, the data capture the information in the previous five years from the data collection period. We used the 2016 NDHS, which is implemented by the new Enumeration Area (EA) under the support of the Ministry of Health (MOH) and funded by the U.S. Agency for International Development (USAID). In the rural areas, the sample is stratified and selected in two stages. In the first stage, wards are selected as the primary sampling units (PSU), with households subsequently chosen from the PSUs. In the urban areas, the sample is nominated in three stages. In the first stage, wards are selected as PSUs; in the second stage, one EA is chosen from each PSU, and finally, households are selected from the EAs. Then data were collected from the women in the reproductive age group within the selected households.

Results

The prevalence of CS in Nepal conforms to the WHO standard with 7.8, 7.5, and 8.1 per 100 deliveries, or 9.8, 8.9, and 9.1 per women’s last births in the previous one, three, and five years, respectively. Older mothers of 30 years old or more, having high incomes, being overweight and obese, using the internet, ante-natal care (ANC) visits of more than four times, ANC by doctors, twin delivery, and having babies of 4 kg or more, had higher odds for a CS while having two or more children seemed to be protective towards CS.

Conclusion

These findings can be used to update health policies surrounding CS delivery to limit unnecessary CS and ensure better health as CS is not without complications.

## Introduction

Caesarean delivery is a primary obstetric lifesaving intervention for a mother and her newborn from pregnancy and childbirth-related complications when undertaken for medical reasons [1-7]. Childbirth through a surgical incision in the mother's abdomen and the uterus is known as caesarean section (CS). Caesarian delivery is also known as C-section or caesarean birth. This surgical performance is often applied when the fetus is unable to be delivered vaginally. Healthcare providers believe it is safer for maternal and perinatal health [8-10]. However, CS is not without risk, and the procedure itself can become life-threatening for both the baby and the mother [2,11-12]. Given this, the World Health Organization (WHO) recommends the appropriate national rate for CS of between 10 and 15 caesarean deliveries per 100 live births [3,12].

Nevertheless, a growing number of children are being born by surgical delivery globally in both developed and developing countries [13-15]. According to data collected from 169 countries, the global rate of CS was estimated to be almost doubled within 15 years, from 16.0 million in 2000 to 29.7 million in 2015, accounting for more than one in five live births [16]. There have been more significant increases in some countries. For instance, in Bangladesh [17], the CS rate increased eight-fold from 2004 to 2017/8 with a four-fold increase in Nepal [12,18] between 2001 and 2011, with this trend continuing [12,17-18]. Previous studies found that CS delivery was higher among urban women, primarily educated women, than rural, uneducated women [9,11,18-22]. As the rate of CS delivery in private hospitals was significantly higher than in government hospitals among South and Southeast Asian women, only women from wealthy groups could afford the high cost and quickly decided for CS [9,23-26].

A recent study showed 40.5% CS in Latin America and the Caribbean region, 32.3% in Northern America, 25% in Europe, and 19.2% in Asia [13]. CS rates in some countries are significantly above the WHO recommendation limit, namely, the Dominican Republic (59.3%), Brazil (56.0%), South Korea (38.0%), and the United States (32.9%) [2,16,27]. Similarly, a study conducted on nine selected developing countries of the South and Southeast Asian region, the highest and third-largest populated regions of the world, reported the highest prevalence of CS in Bangladesh (58.54%), followed by Maldives (33.14%), Pakistan (24.44%), India (18.20%), and Indonesia (21.13%). However, four countries were within the WHO recommended limit, namely, Vietnam (12.27%), Nepal (12.21%), Cambodia (8.12%), and Timor-Leste (7.86%) [8].

The incidence rate for CS is associated with several factors. These include maternal socio-economical determinants incorporating maternal age, residence, education, income, and the number of antenatal care (ANC) during pregnancy and other factors, including body mass index, number of children, the preceding birth interval, and media exposure [28-30]. Maternal age of fewer than 30 years [19-20,31-33] is considered to lower the risk of comorbidities associated with CS [34-35], with advanced maternal age, i.e., 35 years or more, found to significantly increase the rate of CS and adverse outcomes [1,13,30,34,36-37]. With advancing age, the overall rate of CS doubles from 21% (<20 years) to 42% (≥35 years), enhanced by repeated pre-labour CS [28]. Likewise, several studies have consistently found a positive association between advanced maternal age and CS [35,38-40]. Additionally, several studies have documented that as maternal age progresses, significantly above 35 years, the risk of complications increases [19-20,32]. Commonly observed adverse clinical outcomes among pregnant mothers over 35 years include gestational diabetes, preeclampsia, placenta previa, placental abruption, preterm delivery, low birth weight, small-for-gestation-age infants, fetal distress, intrauterine fetal death, and increased perinatal morbidity and mortality [33,40-45].

The mother's residence, especially in low- and middle-income countries (LMICs), appreciably impacts CS rates, increasing mothers in urban areas [9,46]. The level of education also appears to influence CS rates, with typically higher education levels associated with higher CS prevalence rates [1,13,15]. However, this is not always the case, especially in higher-income countries [30,47-49]. Typically, maternal education has a more substantial effect on CS rates than paternal education [47]. Income levels also influence CS rates [9,48,50], alongside maternal obesity [11,13,46]. Mothers of several children are more likely to have a CS with their next child and have adverse consequences [1,51-52].

We are aware that several studies have been undertaken in Nepal regarding maternal services and CS rates. These include studies documenting an increased trend in CS rates in recent years, with Maskey et al. (2019) establishing a rate of 36.8% of total births in their tertiary hospital between 2016 and 2018 [8]. In 2014, Prakash et al. also reported that urban residency, older age, and wealth were associated with higher CS rates [18], with Maru et al. (2016) also documenting that age, location and income influenced care settings for the birth and hence potential CS rates [53]. Similarly, Khanal et al. (2016) showed that while the caesarean delivery rate in their district in Nepal was 14.1% in 2014, overall, this was four times higher in urban versus rural areas at 23.0% vs. 5.8% respectively [21]. Of concern was that prolonged labour and heavy bleeding were common among rural women at home in Nepal. These concerns regarding rural areas were also seen in the study by Karkee et al. (2021), who believed that while great strides have been made in Nepal in recent years to improve care for mothers and reduce maternal mortality, there needs to be an increase in facilities in rural areas offering comprehensive emergency obstetric care to reduce future maternal mortality, which need to be accessible. There also needs to be an increase in maternity waiting homes [54]. Sapkota et al. (2021) also demonstrated the inequality of ante-natal care across the provinces in Nepal [55]. Whilst good progress has been made, there are still gaps requiring tailored investments [55].

More recently, Bhandari et al. (2020) showed that residency, women's education, wealth, and parity were significantly associated with CS rates [12]. In Nepal, CS rates have also been higher among women attending one to three ANC visits [56]. Acharya and Paudel had similar findings with mothers in the highest wealth quintile, with higher education, and delivering in private or NGO-run hospital facilities, having high CS rates above 15% [3]. Overall, women from the highest wealth quintile and those delivering in private facilities were more than three times more likely to deliver by CS versus those in the lowest wealth category and delivering in public facilities [3].

Objectives of the study

We would like to build on these studies by further exploring the prevalence and exploration of the determinants linked to caesarian delivery across all provinces in Nepal. We believe our findings will help provide further insights into the factors affecting caesarian delivery among all seven provinces of Nepal, utilizing the nationally representative samples to provide additional insights. This study will add to, for example, Acharya and Paudel [3] to provide future direction.

## Materials and methods

Data source and study settings

This study analyzed the secondary data from the 2016 Nepal Demographic and Health Survey (NDHS), conducted from June 19, 2016, to January 31, 2017. The survey is undertaken every five years; consequently, the data capture information in the previous five years from the data collection period. Among others, the objective of this survey is to assist in the planning, monitoring, and evaluating of the population health and nutrition programs in Nepal going forward [57].

Study design

We used the 2016 NDHS, which is implemented by the new Enumeration Area (EA) under the backing of the Ministry of Health (MOH) and funded by the U.S. Agency for International Development (USAID) [57]. In the rural areas, the sample is stratified and selected in two stages. In the first stage, wards are selected as the primary sampling units (PSU), with households subsequently chosen from the PSUs. In the urban areas, the sample is nominated in three stages. In the first stage, wards are selected as PSUs; in the second stage, one EA is chosen from each PSU, and finally, households are selected from the EAs. Then, data were collected from the women in the reproductive age group within the selected households.

Outcome variables

The outcome variable of this study was whether the delivery of the women in the previous five years was a CS or not. The variable was coded as ‘Yes’ or ‘No’ for each delivery. A woman might have had more than one delivery in the previous five years, and all these deliveries were considered in calculating CS prevalence rates. The CS prevalence was also estimated per woman based on her latest delivery or the last birth. The previous birth by any woman was also used in assessing the factors associated with a CS in Nepal to avoid redundancies in the factors assessed for multiple deliveries by the same women.

Independent variables

The original 2016 NDHS dataset on the women in the reproductive age group has many variables; however, not all were relevant in assessing the factors associated with CS delivery. These irrelevant variables were excluded from this study and included information on post-natal care and the feeding practices of newborn babies.

The included numerical variables were categorized as explained in the statistical analysis section. The categories were adapted from the available standards and report, if available, including the categorization of BMI, adapted from the WHO guidelines, into ‘normal’ (18.5 to less than 25 kgm^2^), ‘underweight’ (less than 18.5 kgm^2^), or ‘overweight and obese’ (25 kgm^2^ or more); maternal age into ‘less than 25,’ ‘25 to 29,’ or ‘30 years old and more’; and birth weight of the newborn babies into ‘normal’ (2.5 to less than 4 kg), ‘low birthweight’ (less than 2.5 kg), or ‘big baby’ (4 kg and more) [51,58-59]. Other numerical variables, namely, ANC visit was categorized into ‘first,’ ‘second,’ or ‘third’ trimester; total ANC visits into ‘0 to 4,’ or ‘more than 4’ visits; birth interval into ‘0 to 24,’ ‘25 to 48,’ or ‘more than 48’ months; and a total number of children into ‘1,’ ‘2,’ or ‘more than 2’ children.

The poorest and poorer categories of the original wealth index variable were re-categorized into ‘poor’ and richer, and the richest were re-categorized into ‘rich’ for ease of analysis. Frequency of reading, watching television, listening to the radio, and using the internet were re-categorized into ‘no’ (not at all) or ‘yes,’ which includes ‘less than once a week,’ ‘at least once a week,’ and ‘almost every day from the original categories. Other categorical variables were presented following their original categories, namely, the place of residence (‘urban’ or ‘rural’), type of resident (‘usual resident’ or ’visitor’), education level of the women and their husband or partner (‘no education,’ ‘primary,’ ‘secondary’ or ‘higher’ education), gender of the newborn (‘male’ or ‘female’), and whether the newborn is wanted (‘no more,’ ‘then’ or ‘later). The other categorical variables were binomial, which were coded into ‘no’ or ‘yes.’ These included ANC was seen by doctors or nurse/midwives, smoking and history of pregnancy losses, stillbirth, or infant death.

Statistical analysis

The descriptive statistics to illustrate the characteristics of the women recorded in the survey were analyzed using the IBM® SPSS® statistics software (IBM Corp. Released 2020. IBM SPSS Statistics for Windows, Version 27.0. Armonk, NY: IBM Corp). The categorical variables were described using frequency and percentage while the normally distributed numerical variables were summarized using mean and standard deviation (SD). On the other hand, the non-normally distributed skewed variables were summarized using median and inter-quartile range (IQR) because the mean in such variables will be affected by the extreme values in the data. Since the prevalence of CS is analyzed from a categorical variable, frequency and percentage were used, with the addition of its 95% confidence interval (CI) calculated using online software [60].

The data cleaning was undertaken using the IBM® SPSS® statistics software, removing the irrelevant variables before further analysis. The numerical variables were categorized to avoid the problem with the normality assumption since most of these variables were skewed and not normally distributed. Some categorical variables with very small cells were recategorized to balance the number of women in each category and avoid the problem of multicollinearity between the categories.

After data cleaning was completed, the data was saved, and further analysis of simple and multiple logistic regression was performed using the Stata® Statistics/Data Analysis 15.1 software (StataCorp, Texas, USA). As mentioned, the outcome variable for this analysis was the CS deliveries in the 2016 NDHS by the woman selected in the survey based on their last birth within the previous five years. Initially, simple logistic regression analysis was undertaken on all the independent variables, after which the stepwise backward and forward procedures were performed using the cut-off p-value of 0.1. The significant variables were then fitted into the multiple logistic regression to obtain the final model. The non-significant variables were removed and then re-entered and re-removed, if not substantial, one by one into the final model to ensure that all the significant variables were included in the final model.

The variance inflation factor (VIF) values were checked to ensure that there is no multicollinearity problem between the variables and between the categories of the categorical variables in the final model. The final model fitness was then checked by looking at the results of the classification table, the receiver operating characteristic (ROC) curve and area under the ROC curve, the Hosmer-Lemeshow Chi-Square test p-value, and the sensitivity/specificity graph. The results showed that the final model fitness was good, and the results of the multiple logistic regression were reported at the significance level of 0.05 for the 95% CI of the odds ratio (OR).

## Results

Sample characteristics of the respondents

A total of 5038 deliveries were recorded from the 4006 women included in the survey. The median and IQR (in brackets) for the age, the total number of children, total ANC visits, birth interval (in months), and BMI (in kg/m^2^) were 26 (8), 2 (2), 4 (2), 39 (31), and 21.1 (4.5), respectively, while the mean and SD (in brackets) for the birthweight of the newborn babies (in kg) was 3.01 (0.631). The personal categorical characteristics of the women and their ANC characteristics are shown in Tables 1-2. It is noted in the tables that there were some missing values in some of the variables, indicated by double asterisks (**).

**Table 1:** The woman's characteristics in the reproductive age group in the 2016 Nepal Demographic and Health Survey (N = 4006)

Characteristics		Frequency (%)#		
		Normal delivery	Caesarian section	Total
Age (years)				
	< 25	1511 (41.4)	142 (38.8)	1653 (41.3)
	25 - 29	1807 (49.6)	193 (52.7)	2000 (49.9)
	> 30	322 (8.8)	31 (8.5)	353 (8.8)
Type of resident:				
	Usual resident	3394 (93.2)	329 (89.9)	3723 (92.9)
	Visitor	246 (6.8)	37 (10.1)	283 (7.1)
Place of resident:				
	Rural	1562 (42.9)	106 (29.0)	1668 (41.6)
	Urban	2078 (57.1)	260 (71.0)	2338 (58.4)
Education level:				
	No education	1179 (32.4)	52 (14.2)	1231 (30.7)
	Primary	717 (19.7)	46 (12.6)	763 (19.0)
	Secondary	1264 (34.7)	132 (36.1)	1396 (34.8)
	Higher	480 (13.2)	136 (37.2)	616 (15.4)
Husband / Partner’s education: (N=3965)**				
	No education	486 (13.5)	18 (5.0)	504 (12.6)
	Primary	785 (21.8)	55 (15.2)	840 (21.0)
	Secondary	1710 (47.5)	157 (43.3)	1867 (47.1)
	Higher	621 (17.2)	133 (36.6)	754 (19.0)
Wealth index:				
	Poor	1812 (49.8)	67 (18.3)	1879 (46.9)
	Middle	759 (20.9)	63 (17.2)	822 (20.5)
	Rich	1069 (29.4)	236 (64.5)	1305 (32.6)
Body mass index (kg/m^2^): (N = 2032)**				
	Normal (18.5 – <25)	1260 (68.1)	93 (51.4)	1353 (66.6)
	Underweight (< 18.5)	322 (17.4)	17 (9.4)	339 (16.7)
	Overweight (> 25)	269 (14.5)	71 (39.2)	340 (16.7)
Smoking*		241 (6.6)	12 (3.3)	253 (6.3)
Reading*		798 (21.9)	175 (47.8)	973 (24.3)
Watching television*		2180 (59.9)	307 (83.9)	2487 (62.1)
Listening to the radio*		2093 (57.5)	217 (59.3)	2310 (57.7)
Using internet*		550 (15.1)	160 (43.7)	710 (17.7)
#Percentage in the delivery type; **With some missing values; *Values for ‘yes’ only				

**Table 2:** The antenatal care (ANC) characteristics of the woman in the reproductive age group in the 2016 Nepal Demographic and Health Survey (N = 4006)

Characteristics		Frequency (%)#		
		Normal delivery (n = 3640)	Caesarian section (n = 366)	Total (n = 4006)
First ANC visit: (N = 3754)**				
	First trimester	2301 (67.8)	301 (83.1)	2602 (69.3)
	Second trimester	1016 (30.0)	58 (16.0)	1074 (28.6)
	Third trimester	75 (2.2)	3 (0.8)	78 (2.1)
Total ANC visits:				
	0 – 4	2398 (65.9)	136 (37.2)	2534 (63.3)
	> 4	1242 (34.1)	230 (62.8)	1472 (36.7)
Birth interval (months): (N = 2493)**				
	0 – 24	517 (22.2)	21 (13.0)	538 (21.6)
	25 - 48	1037 (44.5)	64 (39.5)	1101 (44.2)
	> 48	777 (33.3)	77 (47.5)	854 (34.3)
Birthweight of the newborns (kg): (N = 2616)**				
	Normal (2.5 – <4.0)	1854 (82.0)	278 (78.5)	2132 (81.5)
	Low birthweight (< 2.5)	263 (11.6)	43 (12.1)	306 (11.7)
	Big baby (> 4.0)	145 (6.4)	33 (9.3)	178 (6.8)
Sex of the newborn:				
	Male	2020 (55.5)	198 (54.1)	2218 (55.4)
	Female	1620 (44.5)	168 (45.9)	1788 (44.6)
Wanted baby:				
	No more	333 (9.1)	13 (3.6)	345 (8.6)
	Then	2873 (78.9)	313 (85.5)	3186 (79.5)
	Later	434 (11.9)	40 (10.9)	474 (11.8)
Total children:				
	1	1302 (35.8)	202 (55.2)	1504 (37.5)
	2	1069 (29.4)	118 (32.2)	1187 (29.6)
	> 2	1269 (34.9)	46 (12.6)	1315 (32.8)
ANC by doctor*		1342 (36.9)	263 (71.9)	1605 (40.1)
ANC by nurse/midwife*		2537 (69.7)	224 (61.2)	2761 (68.9)
Twin pregnancy*		24 (0.7)	6 (1.6)	30 (0.7)
History of pregnancy loss*		862 (23.7)	85 (23.2)	947 (23.6)
History of stillbirth*		45 (1.2)	3 (0.8)	48 (1.2)
History of infant death*		125 (3.4)	10 (2.7)	135 (3.4)
#Percentage in the delivery type; **With some missing values; *Values for ‘yes’ only				

It can be seen from Tables 1-2 that the percentages of visitors, those living in an urban area, higher women’s and husbands’/partners’ education level, wealthy, overweight and obese, reading, watching television, using the internet, first ANC visit in the first trimester, a birth interval of more than 48 months, a large baby, a smaller number of children, ANC by doctors, and twin pregnancy were higher among those with CS deliveries compared to those with normal delivery.

Prevalence of caesarean section delivery

**Figure 1:**
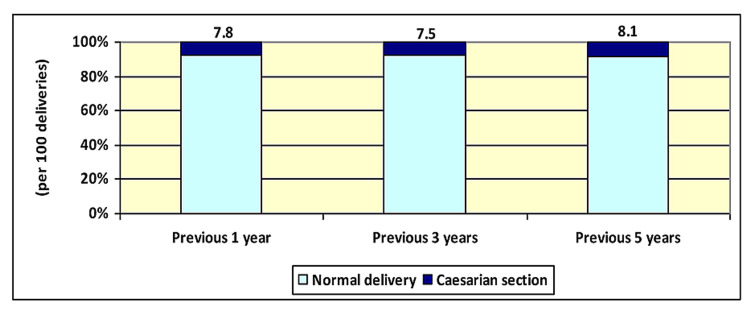
The prevalence of caesarian section based on the total deliveries from the 2016 Nepal Demographic and Health Survey

According to the survey results from the 2016 NDHS, the prevalence of CS delivery in Nepal were 7.8, 7.5, and 8.1 per cent or per 100 deliveries in the previous one, three, and five years, respectively, as illustrated in Figure 1. The detailed results with the 95% CI of the prevalence are shown in Table 3. Out of the 406 CS deliveries recorded in the previous five years, almost half (n = 192; 47.3%) were decided after the labour process began.

**Table 3:** The total number of deliveries, prevalence, and 95% confidence interval (CI) of caesarian section (CS) based on the total deliveries based on the 2016 Nepal Demographic and Health Survey

Duration	No. of total deliveries	No. of CS deliveries*	Prevalence of CS deliveries** (95% confidence interval)
Previous one year	1559	122	7.8 (6.5 – 9.3)
Previous three years	3744	281	7.5 (6.7 – 8.4)
Previous five years	5038	406	8.1 (7.3 – 8.8)
*Out of the total deliveries; **Percent or per 100 deliveries.			

**Figure 2:**
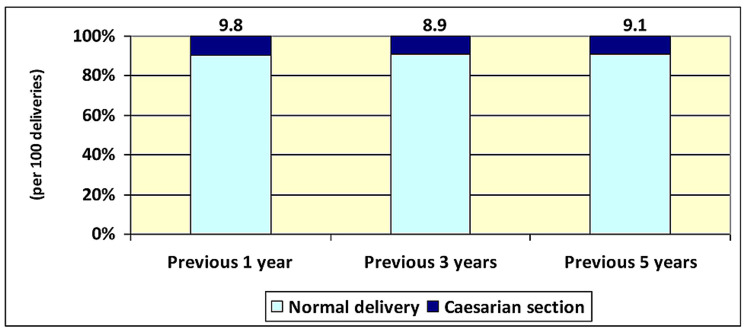
The prevalence of caesarian section deliveries based on the last births of the selected women from the 2016 Nepal Demographic and Health Survey

The prevalence of CS in this survey is shown in Figure 2, with the detailed results and the 95% CI of the prevalence shown in Table 4. It was found that the prevalence of CS delivery in Nepal per 100 last births of the selected women was 9.8, 8.9, and 9.1 in the previous one, three, and five years, respectively. It can be seen that these prevalence rates are higher compared to the prevalence per 100 total deliveries.

**Table 4:** The total number of women, prevalence, and 95% confidence interval (CI) of caesarian section (CS) deliveries based on the last births of the selected women from the 2016 Nepal Demographic and Health Survey

Duration	No. of women	No. of CS deliveries*	Prevalence of CS deliveries** (95% CI)
Previous one year	1062	104	9.8 (8.1 – 11.7)
Previous three years	2761	246	8.9 (7.9 – 10.0)
Previous five years	4006	366	9.1 (8.3 – 10.1)
*Out of last birth of each woman; **Percent or per 100 previous births of each woman.			

Factors associated with caesarean section delivery

The software automatically excluded the cases with missing values during the analysis using the multiple logistic regression, leaving the finalized adequate sample size 1319. The final model obtained from the analysis is presented in Table 5. A total of nine variables were significantly associated with CS delivery among the last birth of the selected women in the previous five years.

**Table 5:** Factors associated with caesarean section delivery from the 2016 Nepal Demographic and Health Survey using multiple logistic regression (N = 1319)

Independent variables		n	Odds ratio (95% CI)	p-value
Age (years):				
	< 25*	604	1.000	-
	25 - 29	426	1.360 (0.892 - 2.076)	0.153
	> 30	289	2.275 (1.346 - 3.844)	0.002
Wealth index:				
	Poor*	478	1.000	-
	Middle	291	1.481 (0.855 - 2.567)	0.161
	Rich	550	1.783 (1.096 - 2.901)	0.020
Body mass index (kg/m^2^):				
	Normal (18.5 – <25)	865	1.000	-
	Underweight (< 18.5)	185	1.043 (0.579 - 1.880)	0.888
	Overweight and obese (> 25)	269	1.773 (1.198 - 2.622)	0.004
Using internet:				
	No*	994	1.000	-
	Yes	325	1.768 (1.217 - 2.568)	0.003
Total ANC visits:				
	0 – 4*	731	1.000	-
	> 4	588	1.663 (1.150 - 2.407)	0.007
Birth weight of baby (kg):				
	Normal (2.5 – <4)*	1061	1.000	-
	Low birthweight (< 2.5)	173	0.668 (0.364 - 1.226)	0.193
	Big baby (> 4)	85	2.454 (1.339 - 4.500)	0.004
Total children:				
	1*	609	1.000	-
	2	412	0.628 (0.417 - 0.946)	0.026
	> 2	298	0.196 (0.096 - 0.400)	<0.001
ANC by doctor:				
	No*	694	1.000	-
	Yes	625	1.723 (1.168 - 2.540)	0.006
Twin pregnancy:				
	No*	1310	1.000	-
	Yes	9	15.917 (3.514 - 72.105)	<0.001
CI = confidence interval; * Reference group; ANC = antenatal care. n=number				

It can be seen from Table 5 that the variable with the largest OR is a twin delivery, which was almost 16 times at higher odds or more likely to have CS delivery as compared to a single pregnancy. However, this should be interpreted with caution because of the small number of twin pregnancies (n=9; 0.7%), which gives rise to a very large 95% CI of the OR (3.514 - 72.105), even though the p-value was highly significant (p<0.001). On the other hand, only one variable was protective towards CS delivery with the OR of less than 1, i.e., having two or more children, which resulted in lower OR than having only one child.

Other statistically significant variables associated with CS delivery in this study were wealth status (the high-income group had 1.78 times higher odds of CS compared to the poor); users of the internet had 1.77 times higher odds of CS compared to non-internet users; those who were overweight and obese had 1.77 times higher odds of CS compared to those with normal BMI; those with five or more ANC visits had 1.66 times higher odds of CS compared to those with less ANC visits, and those whom doctors saw during ANC had 1.72 times higher odds of CS compared to those whom doctors did not see. Older women aged 30 years old and above had higher odds (2.28) of having CS compared to the younger age group of 24 years old and less; large babies with a birth weight of 4.0 kg and above were 2.45 at higher odds of being delivered through a CS compared to babies with a normal birth weight of 2.5 to less than 4.0 kg. The detailed 95% CI of the OR for all significant variables in the final model of the multiple logistic regression is shown in Table 5.

## Discussion

Based on the results from the 2016 NDHS, the prevalences of CS delivery based on the total deliveries in Nepal in the previous one, three or five years were less than 10 to 15 CS per 100 deliveries as recommended by the WHO on the standard of the prevalence of CS delivery [61]. The prevalences based on the last births of the women selected in the survey in the previous one, three or five years were slightly higher, with the highest prevalence of 9.8 per women’s last births during the last year. However, this study's findings did not exceed the standard guidelines established by the WHO.

However, this study did identify nine factors associated with CS delivery in Nepal, which is essential given rising rates [30]. These included older age-group of 30 years old or more, being rich, overweight, and obese, using the internet, ANC visits of more than four times, ANC by doctors, twin delivery, big babies of 4 kg or more, and having more than two or more children.

Overall, older women aged 30 years and more were more likely to have CS than those under 25 years old. A previous study in Nepal showed that CS was the second significant indication for women with a previous CS, which might explain this study’s finding [62]. Multiple studies worldwide have also reported similarly that the mothers’ age remains a significant factor in determining childbirth through CS [21,53-54,63]. Additionally, as discussed, published studies have reported that maternal age (35+ years) is correlated with developing obstetric complications, including death [19-20,32-33,40-45]. These factors were possibly operative in determining a high level of CS seen in this study.

This study shows that the CS delivery rate among women from wealthy households was higher than women from low-income families. This finding is similar to a survey conducted in Ghana that showed that the CS rate was just over one quarter (27.7%) among wealthy women while only 5% among the most disadvantaged group [64-65]. Similarly, a study conducted among 26 sub-Saharan countries, seven countries from South and Southeast Asia, and nine in Latin America and the Caribbean found that CS was substantially lower among impoverished countries [66]. However, another study showed variable results. Whilst there was a decrease in CS deliveries among two African countries, namely, Guinea (from 3.3% to 2.4%) and Nigeria (from 2.9% to 2%) between 1990 and 2014, the CS rate was increased in all the other countries covered by this study [67].

Our study also showed that overweight women were more likely to experience a CS delivery than underweight women. This is similar to a survey conducted in Mongolia, which also found that a higher maternal BMI of women at the first ANC visit had higher odds of CS delivery [68]. A significant association between maternal obesity and CS was also found among Chinese women [23,69-70]. Consequently, being overweight is a predictor of CS delivery and risky for increased fetal and perinatal complications compared to non-obese women [62,71-74]. Excess weight and obesity were also critical factors for emergency CS delivery among migrant women, especially women from sub-Saharan Africa and North Africa [75-76].

Interestingly, in our study, women exposed to and who had been using the internet were more likely to have CS delivery. It may be possible because the internet though provides authentic information and is highly educative, nevertheless, there are a lot of web pages containing misinterpreted data. Often, these fraudulent websites informed that vaginal delivery is extremely painful. This contradicts the findings from an earlier study published in 2007 that reported expected mothers who used the internet found no additional clinical consequences [77]. However, one recent Asian research revealed that pregnant women who used the internet had typically opted for a CS because they learned that vaginal delivery is painful [78]. Similarly, multiple studies involving Asian countries reported that most mothers are in fear of painful vaginal delivery. They believe CS to be a painless, safe, and healthier mode of childbirth, which, moreover, preserve the loveliness of reproductive anatomy.

Consequently, they possess more trust in CS as a child delivery mode [79-81]. This is a concern from now on, with attempts to reduce unnecessary CS rates and, as a result, we are beginning to see healthcare professionals discuss the potential for a vaginal birth trial after a CS, which is likely to grow [1,82].

Of potential concern in our study was that mothers with ANC of more than four visits were at higher odds of having CS delivery than those with fewer visits. This might indicate that the pregnancy was problematic earlier, hence the need for more ANC visits and higher odds of a CS delivery. However, this remains to be seen, although other studies have also shown higher CS rates among women with low ANC visits [83].

In our study, women with a twin pregnancy and those who had big babies of 4 kg were also at much higher odds of being delivered through CS delivery. In Jordan, the CS rate was higher among women with multiple births than singleton births in a certain period [83]. Some big babies are planned to be electively delivered through CS because of the complications that might occur during the normal delivery of these big babies and the comorbidities that might occur during the process.

We also saw that women with two or more children were less likely to have a CS than women who had only one child. This finding contradicts a study in Iran, where the maternal request is considered a fundamental reason for CS. A significant number of women who gave birth four times or more experienced at least one CS delivery [84- 85].

Finally, while the current study descriptively and through simple logistic regression found a higher rate of CS among women living in rural areas and those or their husbands/partners having a higher education level, the multiple logistic regression did not support this.

Article highlights 

CS is a life-saving intervention for pregnant mothers and their babies with obstetric complications. The prevalence of CS delivery in Nepal based on the 2016 NDHS was lower than the WHO's recommended rate of 10 to 15 CS per 100 live births. In this study, the personal characteristics found to have significantly higher odds of having CS delivery were those in the older age group (> 30 years old), being rich, overweight, and obese, and using the internet. The ANC characteristics found to be significantly more likely to have CS delivery in this study were those with ANC visits of more than four times, ANC by doctors, twin delivery, and big babies of 4 kg or more. Having two or more children seemed to be protective towards CS delivery in this study.

Limitations of the study

This cross-sectional design prevents any causal relationship from being confirmed in this study; hence, it is recommended to do a cohort study in the future for such a conclusion. The presence of many missing data in some of the critical variables, such as BMI and birth weight of the newborns, might well have affected the findings regarding associated factors for CS delivery. Despite these limitations, we believe our findings are robust, providing direction for future health policy and planning.

Recommendation

Expecting mothers and women planning for pregnancy should be educated about the benefits of vaginal delivery and the adverse effects of CS. Common people need to be aware that CS is not a one-step-ahead alternative or a preferable choice over vaginal delivery. Instead, CS is only to be opted for when vaginal delivery becomes life-threatening for the mother or baby or both. Monitoring authorities need to ensure pregnant women are not influenced by medical staff to agree to unnecessary CS. Adoption of a healthy lifestyle should also be encouraged in the population.

## Conclusions

CS is a means of delivery that should be applied only when absolutely indicated, and a trained health care provider should assess the need for CS and be performed when deemed safer for maternal and fetal health. The World Health Organization has recommended the appropriate national rate for CS to be 10-15 cesarean deliveries per 100 live births. This study shows that the prevalence of CS in Nepal conforms to the guideline by the WHO. However, the study's findings have led us to suggest that the rate of CS can be curtailed further by taking some measures with respect to modifiable risk factors. Thereby, this study advocates the need of building a high level of awareness among people concerning the safety of normal vaginal delivery and promote strict regulatory measures associated with an increased rate of CS. It is hoped that the results of this study can advocate safer and less CS delivery among pregnant mothers in the future.
